# Optimization of Preservation Methods Provides Insights into Photosynthetic Picoeukaryotes in Lakes

**DOI:** 10.1128/spectrum.02557-21

**Published:** 2022-05-12

**Authors:** Changqing Liu, Jin Lei, Min Zhang, Fan Wu, Mingdong Ren, Jinsheng Yang, Qinglong Wu, Xiaoli Shi

**Affiliations:** a State Key Laboratory of Lake Science and Environment, Nanjing Institute of Geography and Limnology, Chinese Academy of Sciences, Nanjing, China; b University of Chinese Academy of Sciences, Beijing, China; c Jiangsu Collaborative Innovation Center of Regional Modern Agriculture and Environmental Protection, Huaiyin Normal University, Huaiyin, China; Nanyang Technological University

**Keywords:** photosynthetic picoeukaryotes, flow cytometry, preservation methods, Pluronic F68

## Abstract

As the key contributor to plankton biomass and nutrient cycling in aquatic ecosystems, photosynthetic picoeukaryotes (PPEs) have been recently investigated in freshwater ecosystems. However, the limited access to remote areas creates challenges for PPE sample preservation before sorting and counting by flow cytometry (FCM) in the laboratory. Here, we explored the effects of different preservation methods on the PPE community by combining FCM sorting and high-throughput sequencing. Our results showed that dimethyl sulfoxide (DMSO) cryoprotection could destroy the fluorescence and cell structure of the PPEs, making the subsequent FCM analysis and sorting difficult. Aldehyde fixation maintained the PPE fluorescence, and the fixed samples were of sufficient quality for abundance analysis and sorting by FCM. However, the sequencing results showed that, after preservation by aldehydes, the proportion of PPEs dramatically decreased to approximately 10%, in comparison to 90% in the fresh samples, and the sequences of Ascomycota significantly increased. In contrast, preservation with Pluronic F68 (F68) not only could maintain the PPE abundance close to the initial value but also could keep the PPE community similar to that in the fresh samples over a storage time of 6 months. Thus, F68 cryopreservation is a suitable preservation method for PPE communities from freshwater lakes.

**IMPORTANCE** PPEs contribute significantly to primary productivity in freshwater ecosystems. The combination of FCM sorting and high-throughput sequencing has been shown to be a powerful approach and can largely improve our view of the PPE diversity. However, the water samples could not be counted and sorted immediately after sampling from many lakes due to the inaccessibility of FCM in the field. Thus, the comparison of different preservation methods that allow subsequent analysis of the community structure by high-throughput sequencing is an urgent need. Our results indicated that F68 cryopreservation could maintain the PPE abundance close to the initial value and keep the community similar to that in the fresh samples over a storage time of 6 months.

## INTRODUCTION

As the key contributor to plankton biomass and nutrient cycling in aquatic ecosystems, autotrophic picophytoplankton (<3 μm), including photosynthetic picoeukaryotes (PPEs) and picocyanobacteria (PCY), plays an important role in aquatic ecosystems under global warming (1–3). Compared with PCY, PPEs have much more complex community structures and functions, and they are abundant in eutrophic lake ecosystems, such as Lake Chaohu and Lake Taihu (4). Flow cytometric (FCM) sorting and high-throughput sequencing have been used to investigate picophytoplankton community structures in recent years (5, 6). Early relevant studies were focused on marine ecosystems (2, 3, 7), but most water samples could not be immediately analyzed unless the sampling ship was equipped with FCM (8). Recently, with the increasing knowledge that PPEs contribute significantly to primary productivity in freshwater ecosystems, investigations of the PPE community structure have been carried out in different lake ecosystems (5, 9). However, FCM analysis of lake samples would be challenging, due to the inaccessibility of FCM during the lake survey. Before long-term transport, the samples need to be preserved to maintain their community structure until they arrive at the laboratory. Therefore, identification of a proper preservation method is an urgent need to maintain the consistency of the PPE community between fresh and preserved samples.

As a preservation method, fixation alone or combined with cryopreservation is commonly employed in PPE research (8, 10, 11). Aldehydes, such as formaldehyde (FA) and glutaraldehyde (GA), are widely used as fixation reagents for FCM samples (12–14). Microbial samples are usually preserved immediately upon collection by snap-freezing, which minimizes handling and exposure of the sample to preservation artifacts (15, 16). Samples for FCM are generally snap-frozen in liquid nitrogen and stored at −80°C until analysis after fixation (8, 17). To avoid cellular damage during cryopreservation and subsequent thawing, cryoprotective agents, such as dimethyl sulfoxide (DMSO) (18) and Pluronic F68 (F68) (14), have been widely applied in microbial research. However, those preservation approaches were focused on maintaining the autofluorescence of PPEs for FCM analysis, and investigators did not evaluate their potential effects on the PPE community structure.

In this study, we investigated the effects of seven preservation methods on PPE community structure by combining FCM sorting and high-throughput sequencing. The purpose of this study was to identify a proper protocol to keep the PPE community structure of preserved samples as close as possible to that of fresh samples, which is very important for studying the structure and function of PPEs in freshwater lakes.

## RESULTS

### Effects of preservation methods on PPE abundance.

**FIG 1:**
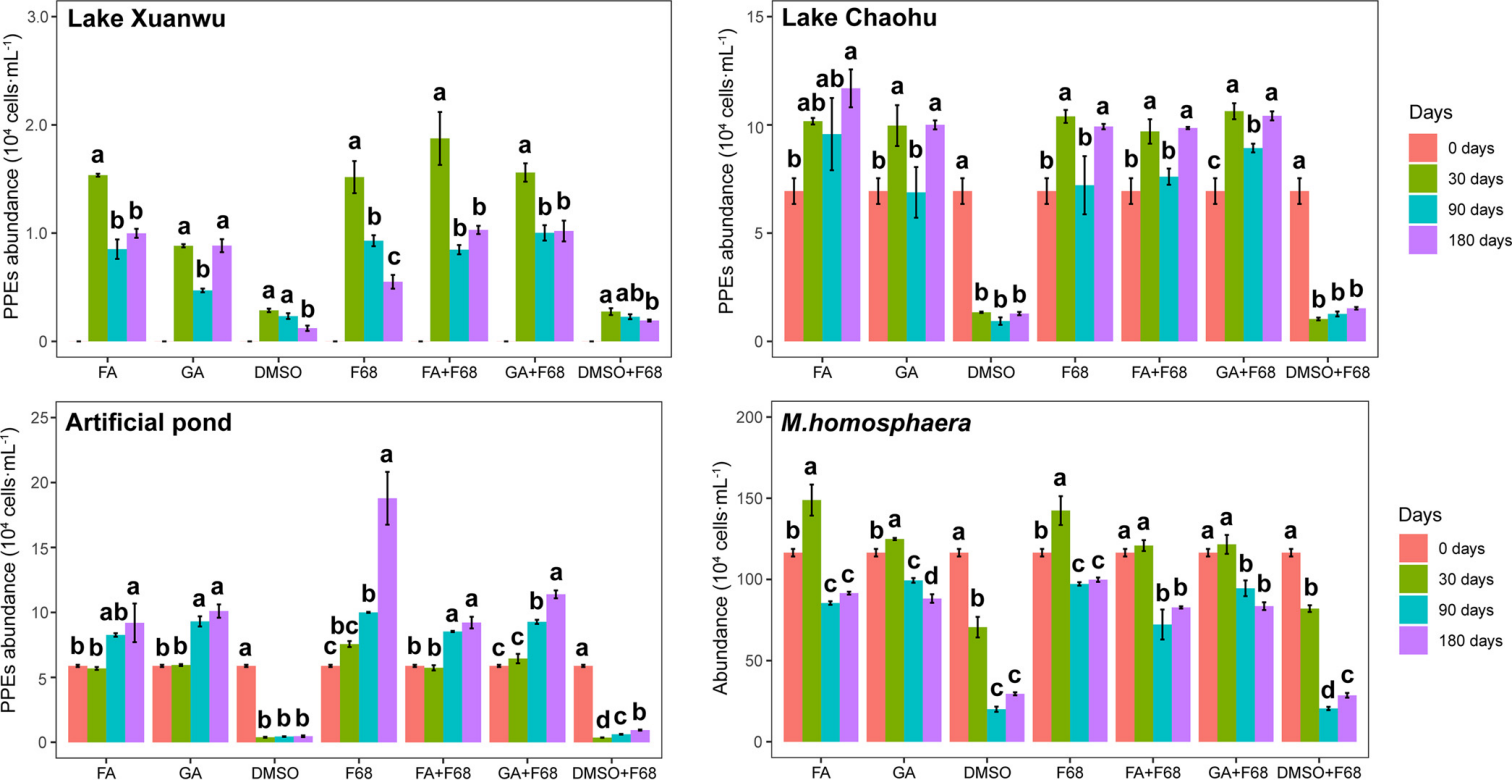
PPE abundance in different lakes and cultures during storage with the different preservation methods. Different lowercase letters indicate significant differences between treatments, as revealed by one-way ANOVA with Duncan’s multiple-range test at *P* < 0.05. The 0 days samples are fresh samples from each lake without preservation. Due to the absence of records, the PPE abundance in the fresh samples from Lake Xuanwu is missing.

Although the PPE abundance of the fresh samples from Lake Xuanwu was missing, a relatively low abundance of PPEs was observed after DMSO and DMSO plus F68 preservation. For samples from Lake Chaohu, the artificial pond, and Mychonastes homosphaera cultures, the PPE abundance sharply decreased after DMSO and DMSO plus F68 preservation but seemed to increase after other preservation methods. Additionally, the change of PPE abundance with storage time after non-DMSO preservation was different between the M. homosphaera culture and lake samples. PPE abundance declined after 30 days for pure culture samples but fluctuated, with relatively high abundance, for lake samples (Fig. 1). In order to assess whether PPE cells would separate from tube wall after preservation, fluorescence microscopy was used to observe the PPE cells on the inner surface of tubes. After F68 preservation, few cells could be observed on the side of the tube containing M. homosphaera culture samples (see Fig. S1 in the supplemental material).

### OTU distribution patterns revealed by high-throughput sequencing of the sorted samples.

The PPE community at 0 days (fresh samples), 30 days, 90 days, and 180 days was investigated by combining FCM sorting and high-throughput sequencing. The sequencing of the 18S rRNA gene yielded 13,327,068 high-quality sequences and 2,422 operational taxonomic units (OTUs), at a 97% similarity level, from 113 samples. Due to the massive bias of fluorescence signal from the standard distributions (see Fig. S2), the PPEs were barely identified and sorted from the preserved samples after DMSO and DMSO plus F68 preservation. Therefore, there were no high-throughput sequencing results for these samples.

The PPE community of fresh samples showed high levels of diversity and possessed a different taxonomic composition (see Table S1). In Lake Xuanwu, read numbers of the PPEs (89.59% of the total reads) were far greater than those of nonpigmented eukaryotes (non-PPEs), although they possessed similar OTU richness. Chlorophyta represented a majority of the PPE diversity (71 OTUs) and also contributed the greatest abundance in Lake Xuanwu (43.66%). Bacillariophyta also contributed great abundance (40.31%) in Lake Xuanwu, although they represented only 11 OTUs. However, Bacillariophyta was the most abundant phylum (83.70%) in Lake Chaohu despite the relatively low diversity. Similar to Lake Xuanwu, Chlorophyta dominated in the artificial pond according to diversity (70 OTUs) and abundance (21.74%). The other PPE groups, including Chrysophyceae, Dinophyceae, Eustigmatophyceae, Haptophyceae, Katablepharidophyta, Raphidophyceae, Synurophyceae, and Xanthophyceae, also contributed to the high level of diversity in those lakes despite their relatively low abundance (see Table S1).

### Effects of preservation methods on the PPE community composition.

**FIG 2:**
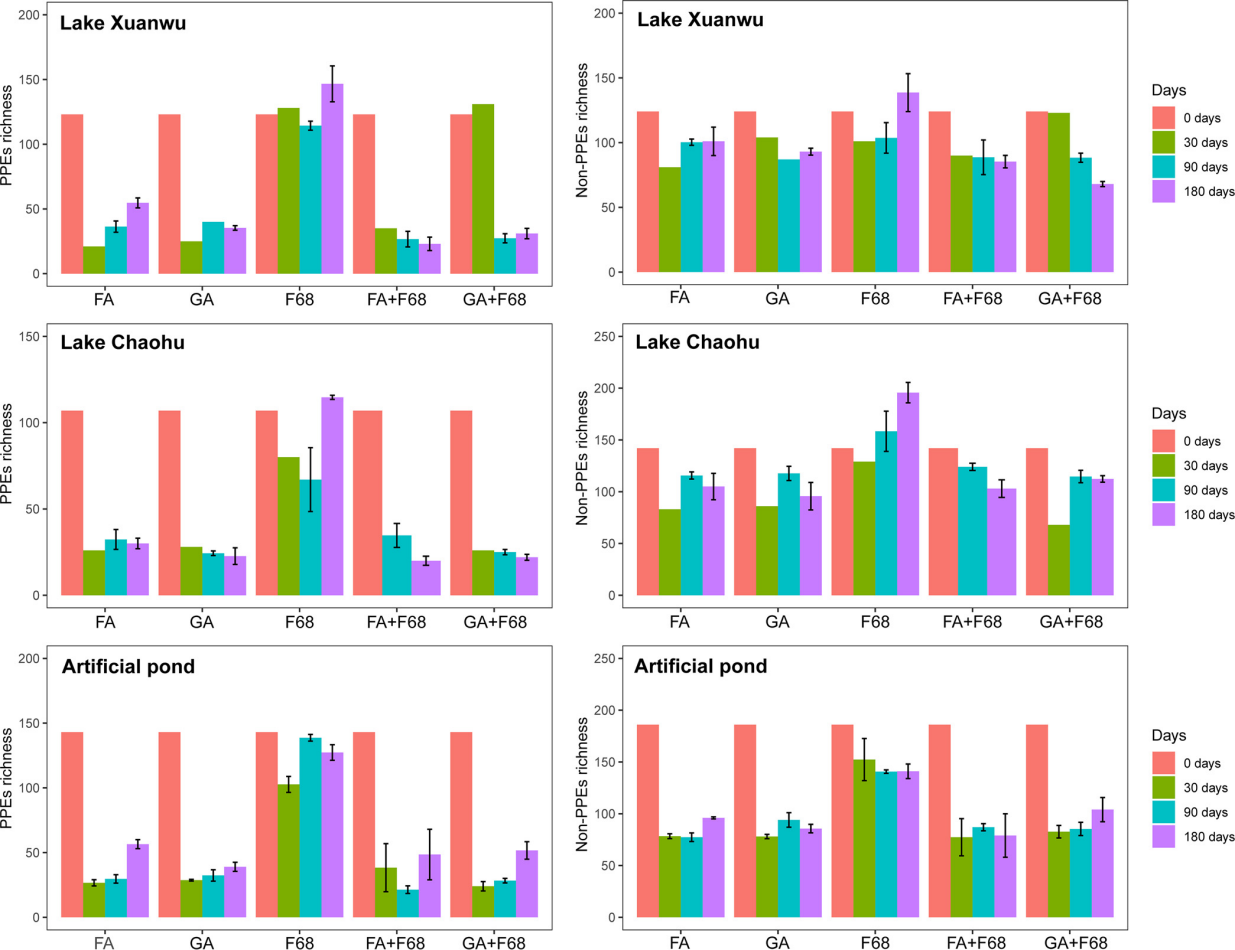
OTU richness of PPEs (left) and non-PPEs (right) in different lakes during storage with the different preservation methods. The 0 days samples are fresh samples from each lake without preservation.

PPE richness did not change considerably after F68 preservation, compared with that of the fresh samples (*t* test, *P* > 0.0001), but it significantly decreased with the other fixation methods in all of the lakes (*t* test, *P* < 0.0001) (Fig. 2). Notably, compared with the non-PPEs, the decrease in PPE richness was larger (Fig. 2).

**FIG 3:**
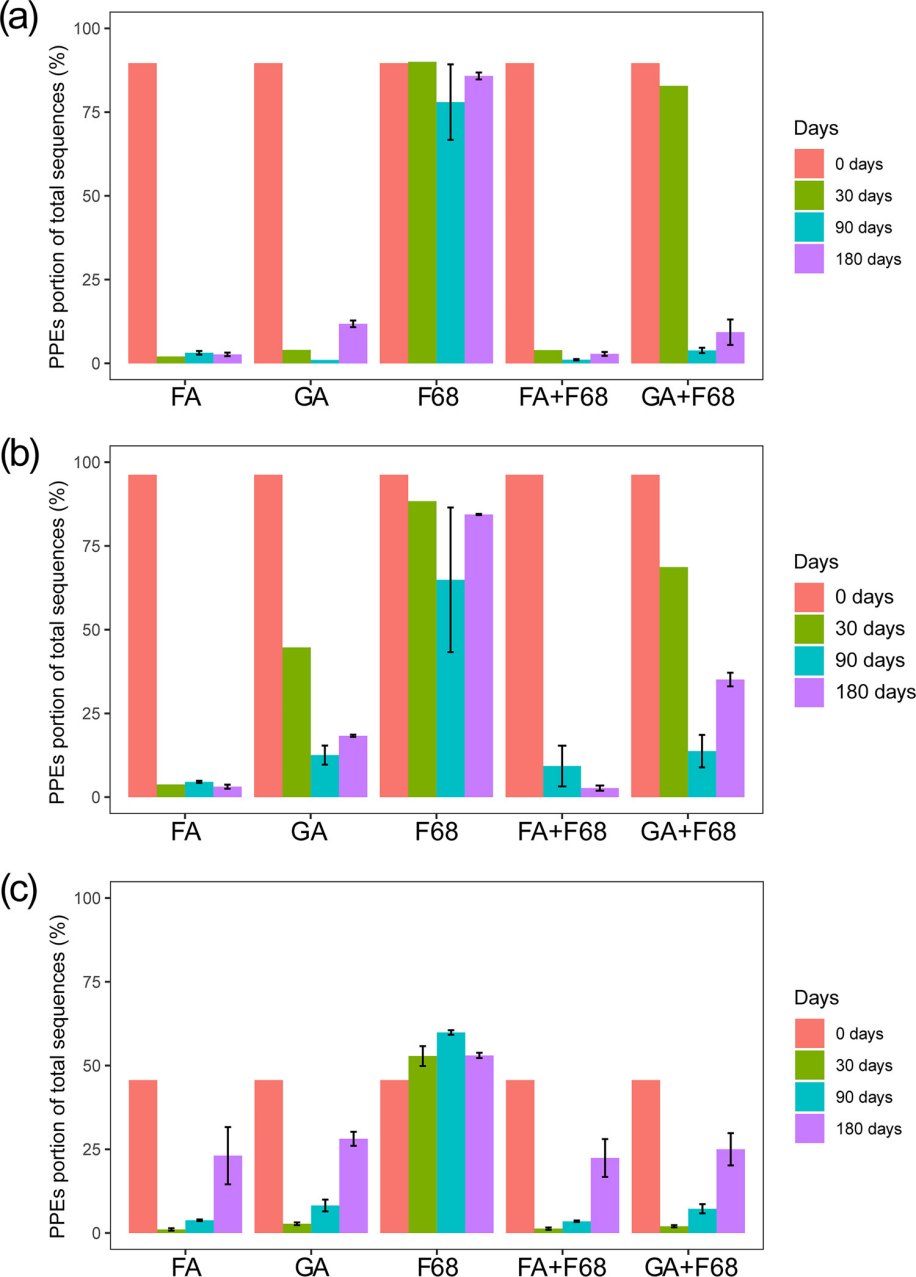
PPE proportions of the total sequences for Lake Xuanwu (a), Lake Chaohu (b), and the artificial pond (c) during storage with the different preservation methods. The 0 days samples are fresh samples from each lake without preservation.

**FIG 4:**
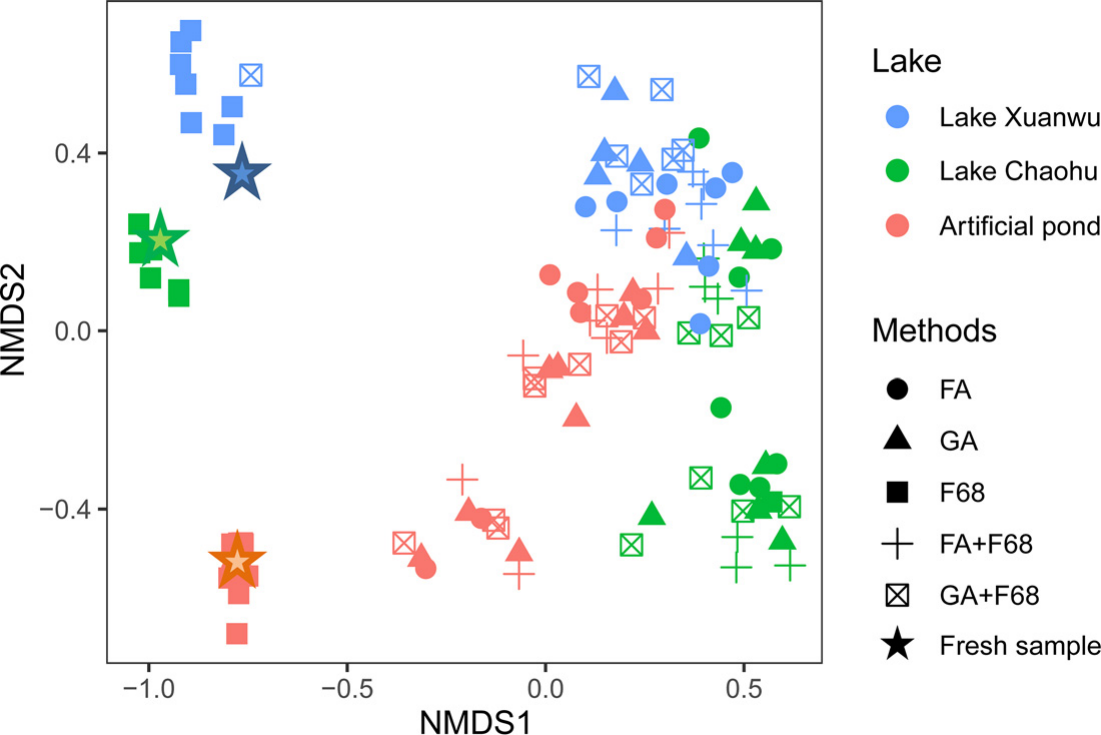
NMDS analysis of the PPE community during storage with the different preservation methods based on Bray-Curtis dissimilarity. The colors represent the lake types, and the shapes represent the preservation methods.

**FIG 5:**
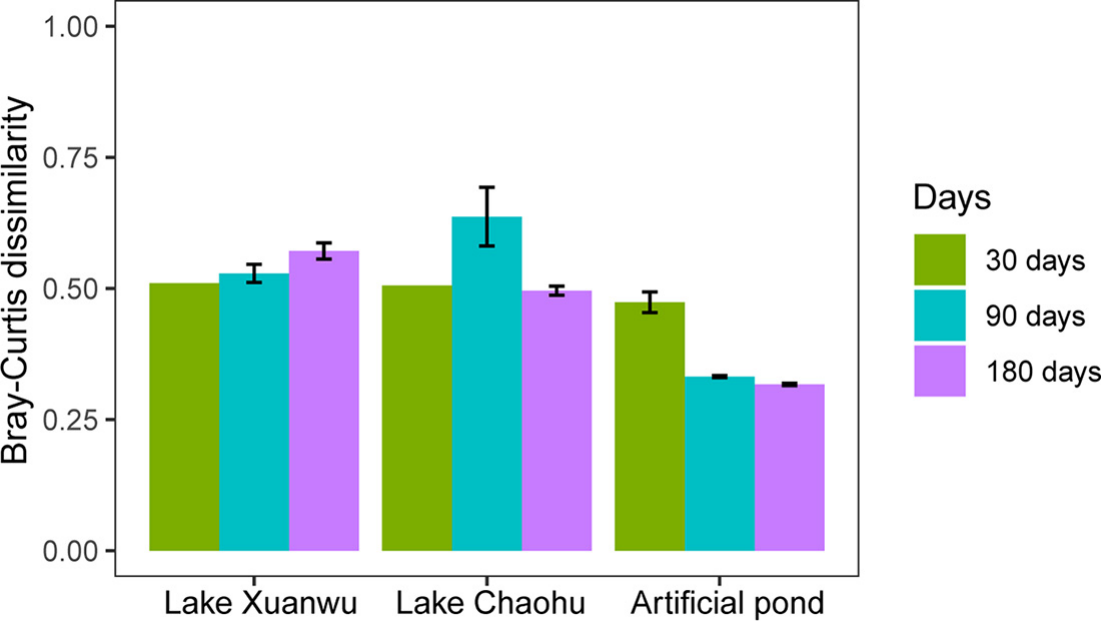
Bray-Curtis dissimilarity between the fresh and F68 preservation samples during storage.

Compared with the fresh samples, the PPE portion in the preserved samples significantly decreased with all preservation methods (*t* test, *P* < 0.0001) except for F68 preservation (Fig. 3). The PPE portion decreased from 90% in Lake Xuanwu, 96% in Lake Chaohu, and 46% in the artificial pond to below 10% after preservation. Conversely, the proportions of PPEs in F68 preservation samples were close to those of fresh samples and had only slight changes after a storage time of 6 months, being 86% for Lake Xuanwu, 84% for Lake Chaohu, and 53% for the artificial pond. The nonmetric multidimensional scaling (NMDS) ordination showed that only the PPE community in the F68 preservation samples could be clustered with that in the fresh samples (Adonis test, *P* < 0.0001) (Fig. 4), and Bray-Curtis dissimilarity for the PPE community between the fresh and F68 preservation samples was not significantly different during storage (Kruskal-Wallis test, *P* > 0.0001) (Fig. 5).

**FIG 6:**
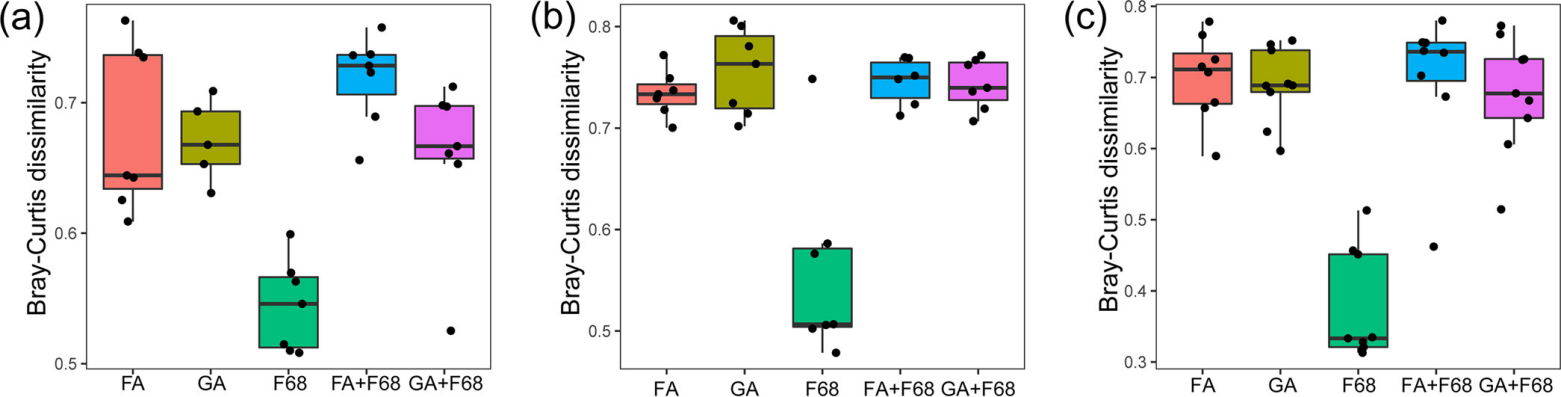
Bray-Curtis dissimilarity between the fresh and preservation samples for Lake Xuanwu (a), Lake Chaohu (b), and the artificial pond (c).

However, the PPE communities from samples with other preservation methods were clustered together, being significantly different from that of the fresh samples from all lakes (Adonis test, *P* < 0.0001) (Fig. 4). Bray-Curtis dissimilarity of the PPE community for each sample with various preservation methods and the fresh samples showed that the dissimilarity was significantly lower in the samples after F68 preservation than in those after the other preservation methods for all lakes (Kruskal-Wallis test, *P* < 0.0001) (Fig. 6). Although the non-F68 preservation samples could be divided into three clusters based on the lakes (Fig. 4), the storage time also affected the community structure in each lake (see Fig. S3). The PPE community of the non-F68 preservation samples clustered according to storage time rather than the preservation method for all lakes (see Fig. S3), and Bray-Curtis dissimilarity for each pair of lakes significantly increased with storage time (Kruskal-Wallis test, *P* < 0.0001).

**FIG 7:**
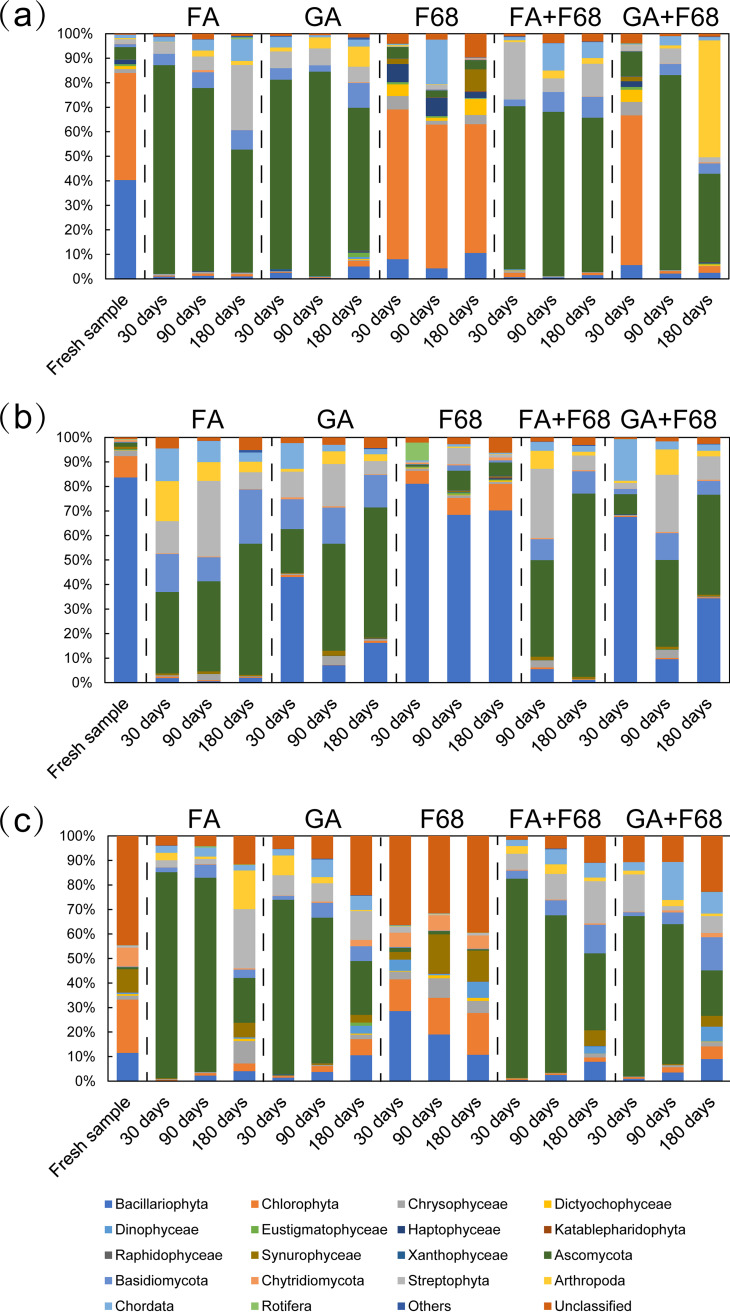
Composition of the PPE communities for Lake Xuanwu (a), Lake Chaohu (b), and the artificial pond (c) during storage with the different preservation methods.

Furthermore, Chlorophyta and Bacillariophyta dominated the fresh samples for all lakes, and then the dominant taxon was replaced by Ascomycota in samples with all preservation methods except F68 preservation (Fig. 7). Compared with Chlorophyta, Bacillariophyta could maintain a certain abundance after non-F68 preservation. However, fluctuations in the proportion of the main group of PPEs were inevitable even after F68 preservation.

### Responses of PPE taxa to the preservation methods.

The shift in the PPE community was caused by the different responses of the PPE and non-PPE taxa to the preservation methods. The F68 preservation samples across all storage times maintained most of the OTUs retrieved from the fresh samples (see Fig. S4). However, some OTUs that were not detected in the fresh sample became detectable after preservation. Except for F68 preservation, most PPE OTUs disappeared after preservation for all lakes (see Fig. S5).

**FIG 8:**
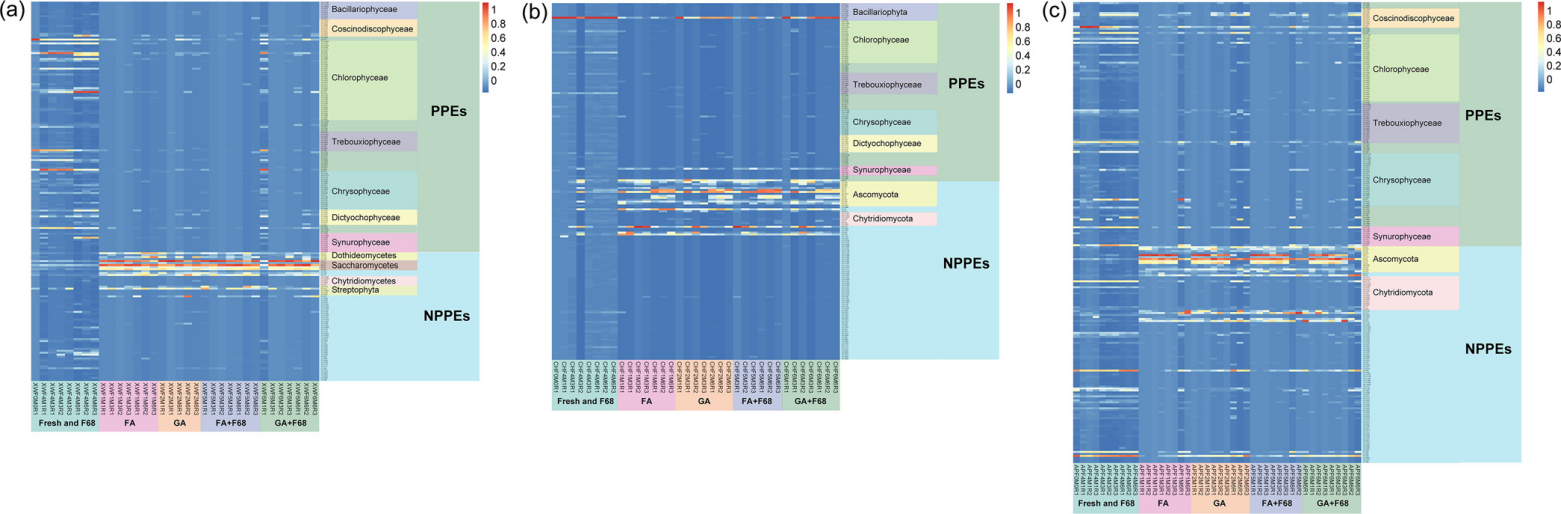
Relative abundance distributions of the indicator OTUs for Lake Xuanwu (a), Lake Chaohu (b), and the artificial pond (c) with the different preservation methods.

The indicator OTUs had similar taxonomic compositions over the lakes, with numbers ranging from 195 OTUs for Lake Xuanwu and 184 OTUs for Lake Chaohu to 232 OTUs for the artificial pond (Fig. 8). Those OTUs with close phylogenetic affiliations showed similar responses to the preservation methods. Among the main PPE taxa, Bacillariophyta, including Bacillariophyceae and Coscinodiscophyceae, Chlorophyta, including Chlorophyceae and Trebouxiophyceae, and Chrysophyceae, Dictyochophyceae, and Synurophyceae decreased sharply after preservation. As the dominant non-PPE taxa, Ascomycota, especially Dothideomycetes and Saccharomycetes, were abundant after preservation. In addition, some other non-PPE taxa, such as Chytridiomycota and Streptophyta, increased slightly after preservation.

## DISCUSSION

### Aldehyde fixation is not optimal for PPE community analysis.

The effects of the common preservation methods on PPEs were shown in previous research; however, those studies were focused on PPE abundance while ignoring subsequent molecular biology analyses (14, 19). The common preservation methods usually combine aldehyde fixative with or without cryoprotectants. Our results indicated that PPE abundance could be maintained close to that in the fresh samples after fixation alone (FA and GA) or with F68 cryoprotectants (FA plus F68 and GA plus F68). Cell lysis and fluorescence intensity determined the counting results of FCM (13); therefore, aldehydes may efficiently preserve the cellular structures and the pigment autofluorescence of PPEs (20). However, the responses of PPE abundance to aldehyde (FA and GA) preservation are inconsistent between pure culture and lake samples. Unlike the single cells of pure cultures, algal colonies widely exist in lakes. Aldehydes may damage extracellular proteins and lead the algal colony to disperse, resulting in an increase in PPE abundance. However, the impact of aldehydes on natural lake samples may be difficult to predict, because the responses of PPE to aldehydes are often species dependent (14).

Aldehydes were valid fixatives for the analysis of PPE abundance; however, the PPE community can change substantially in composition after aldehyde fixation with or without F68 cryoprotection. This might be caused by DNA damage of the PPEs after aldehyde fixation. Although fixation with aldehydes has recently become widely used in microbiome research (21–23), damage to DNA by aldehydes remains inevitable (24, 25). To maintain cellular and organelle structures, aldehydes such as FA and GA cross-link and coagulate proteins, consequently trapping the DNA in the cell and making the DNA unavailable for PCR amplification (26, 27). Therefore, the results of PPE high-throughput sequencing would be dramatically biased due to unsuccessful DNA amplification after aldehyde fixation. However, the combination of FCM sorting and high-throughput sequencing has been shown to be a powerful approach and can largely improve our view of the PPE diversity. The data based on sequencing could present only the relative composition of PPEs. The PPE proportion was approximately 10% after aldehyde fixation, which is much lower than that in the fresh samples. Furthermore, different microbial groups showed diverse responses to the preservation methods (15, 28). Compared with Chlorophyta, Bacillariophyta could maintain a certain abundance after aldehyde fixation, which may be due to their unique cell walls incorporating silica (29).

In contrast to the PPEs, fungi, especially Ascomycota, could dominate in the sequencing database, contributing approximately 90% after aldehyde preservation. Compared with other fungi, Ascomycota are usually the predominant fungi in lakes (30, 31) and have high probability to attach PPEs and then to be sorted out by FCM. The dominance of Ascomycota implied that the damage from aldehydes on PPEs is more serious than that for Ascomycota, probably due to their different cell wall components (29, 32). Because of the lack of photosynthetic pigments, Ascomycota sequences obtained from sorted PPE samples are mainly from fungal spores that are directly attached to PPE cells (33). In fact, most OTUs belonging to Ascomycota could be detected in all lakes; thus, the proportion of Ascomycota OTUs could be used as an index to evaluate the degree of deviation of preserved PPE samples from fresh samples.

### F68 cryopreservation is a proper preservation method for PPE community investigation.

Although aldehyde fixation would damage PPE DNA, there is little debate regarding whether cryopreservation is still an effective preservative for phytoplankton DNA (18, 34, 35). Maintaining samples at ultracold temperatures has become practical due to the widespread use of portable liquid nitrogen containers and car refrigerators in the field. However, fast freezing would increase the risks of cellular injuries due to intracellular ice formation (36, 37). Thus, cryoprotectants, such as DMSO and F68, are usually applied for cryopreservation methods (38, 39).

A rapid decrease in PPE abundance was observed after DMSO and DMSO plus F68 cryoprotection, and these methods could not satisfy the requirements for further molecular biology analyses. Although DMSO is effective for algal preservation in some cases (39), it distorts the morphological characters (22) and fluorescence (40) of the preserved species. The sorting and counting of PPEs by FCM are based on their cellular fluorescence signals and properties (41). In our study, the biases of fluorescence signals were observed in the counting and sorting of the preserved samples after DMSO and DMSO plus F68 preservation (see Fig. S2 in the supplemental material), which implied the inefficiency of PPE counting and sorting. This result means that DMSO is not a proper cryopreservation reagent for PPE communities.

F68 cryoprotection could maintain the relative stability of PPE abundance during storage. Interestingly, the PPE abundance with F68 cryoprotection was greater than that in the fresh lake samples. As a nonionic surfactant (38), F68 could improve the separation of the PPE cells from the tube walls, leading to relatively greater cell abundance than in the samples without added surfactant. As a surfactant, F68 adsorbs onto cell surfaces and then interacts with their cytoplasmic membranes to reduce the damage of freeze-thawing procedures (42), which would reduce PPE cell loss during cryopreservation. However, the responses of PPEs to F68 may be species dependent (14). Thus, the changing patterns of PPE abundance after F68 preservation may be different for various lake samples. In addition, F68 is used as a thickener, antifoaming agent, dispersant, and emulsifier, which reduces the shear stress of cell membranes (43–45). These cell surface interactions mean that F68 does not directly interact with cellular DNA and may maintain the DNA integrity of PPEs. Moreover, F68 is a low-cost and nontoxic agent that is environmentally friendly and harmless to humans. Our results indicated that F68 maintained not only the stability of PPE abundance but also the characteristics of the PPE community during a storage time of 6 months; therefore, F68 cryopreservation is the proper preservation method for PPE communities from freshwater lakes. Considering the cryopreservation of PPE cells from marine water (14), F68 could also be a potential preservative for salt lake samples, which requires further exploration.

We found that F68 cryopreservation tended to recover a greater diversity of PPE taxa than other preservation methods, even for some taxa that were not detected in the fresh samples. Artificial methodological biases, such as chimeras generated by PCR and artificial taxa originating from *de novo* OTU binning algorithms, may contribute to this greater diversity (46). The other cryoprotectants, i.e., glycerol and glycine betaine, are also preservatives for FCM analysis (47, 48). However, glycerol cryopreservation could underestimate PPE abundance due to unavoidable and unquantifiable cell loss (49), and glycine betaine cryopreservation yielded a small number of cells for the samples from a hypersaline lake (47). Thus, both of these cryoprotectants could not satisfy the requirements of large-scale lake surveys.

## MATERIALS AND METHODS

### Sampling and preservation.

From January to March 2019, we collected samples from three freshwater lakes, namely, Lake Xuanwu (an urban shallow lake [32°4′32.36″N, 118°47′32.27″E]), Lake Chaohu (a eutrophic shallow lake [31°30′45.92″N, 117°31′13.42″E]), and an artificial pond at the Nanjing Institute of Geography and Limnology, Chinese Academy of Sciences (32°3′39.38″N, 118°48′0.06″E). Surface, intermediate, and bottom water samples were collected and mixed to obtain water samples representing the whole water column. The freshwater samples were stored in sterile bottles and transported immediately to the laboratory within 3 h, on ice in the dark. Fresh samples were analyzed using a FACSJazzSE FCM system (Becton, Dickinson, San Jose, CA, USA). The preprocessing, counting, and sorting procedures for FCM were described in detail in our previous study (5, 50). Briefly, 3-μm standard beads (SPHERO URFP-30-2, 3.0 μm; Spherotech, USA) were first used for the size control. On the basis of different pigment autofluorescence, two groups of picophytoplankton cells were clearly distinguished by FCM and detected with the highest frequencies of occurrence during the investigation. PPEs were characterized by greater forward scattering (FSC) and rich far-red fluorescence from chlorophyll a. Preservation samples were subjected to seven preservation methods, i.e., 2% FA, 0.25% GA, 0.01% F68, 10% DMSO, 2% FA plus 0.01% F68, 0.25% GA plus 0.01% F68, and 10% DMSO plus 0.01% F68. After standing at room temperature protected from light for 15 min, the samples were quickly frozen in liquid nitrogen and stored frozen at −80°C. The preserved samples after 30 days, 90 days, and 180 days of storage were analyzed using a FACSJazzSE FCM system (Becton, Dickinson). The preserved samples were analyzed three times for each method at different storage times (excluding the samples collected from Lake Xuanwu and Lake Chaohu at 30 days), and the concentration for the preservation methods was taken as the final concentration. After FCM analysis, the PPE cells were sorted into sterile centrifuge tubes and stored at −20°C until DNA extraction was performed. We used sterile phosphate-buffered saline (PBS), which is a common suspension buffer for cell sorting by FCM, as the negative control. The PBS solution was supplemented by preservatives and was analyzed using a FACSJazzSE FCM system. No PPE cells were detected, which confirmed the absence of contamination (see Fig. S6 in the supplemental material).

### Fluorescence microscopy observations of pure PPE cultures.

M. homosphaera, the dominant eukaryotic picophytoplankton in most eutrophic lakes (4, 51), was chosen as a single PPE culture. The M. homosphaera culture isolated from Lake Chaohu (52) was preserved in a fashion similar to the field sample and analyzed by FCM. In order to assess whether PPE cells would separate from the tube wall after preservation, the tube that contained M. homosphaera was emptied and manually broken into pieces, and the inner walls were observed by fluorescence microscopy at ×400 magnification (Axio Scope A1; Zeiss, Germany).

### DNA extraction, PCR amplification, and MiSeq high-throughput sequencing.

DNA was extracted from the sorted samples using the DNeasy blood and tissue kit (Qiagen) for subsequent target fragment amplification and high-throughput sequencing. The DNA extraction was performed according to the kit instructions. PBS solution was used as the negative control, and there was no band on the gel (see Fig. S7). The V4 regions of the 18S rRNA genes were amplified using the universal eukaryote primers Ek-NSF573 (5′-CGCGGTAATTCCAGCTCCA-3′) and Ek-NSR951 (5′-TTGGYRAATGCTTTCGC-3′), which were selected by an *in silico* approach because they allowed for the best recovery of richness from the freshwater small eukaryote sequences available in public databases (53). PCR amplification was performed using a touchdown program, as described previously (33). The PCR products were purified using a PCR purification kit (Agencourt AMPure XP; Beckman) according to the manufacturer’s instructions. The amplicons were then sent for sequencing on an Illumina MiSeq 250PE platform at the Center for Genetic and Genomic Analysis (Genesky Biotechnologies Inc., Shanghai, China).

### PPE sequence analysis.

Cutadapt was used to truncate reads at the tail primer (54), and then the terminal bases of the paired reads with low quality scores (scores of <Q15) were eliminated by the FastX-Toolkit. FLASH was then used to merge the paired reads (55). All of the merged reads were further cleaned and filtered against the quality criteria using USEARCH v10 (56). The retained reads from each sample were pooled, dereplicated, and clustered to define the OTUs using the cluster_otus algorithm with a 97% similarity threshold. Representative sequences of each OTU were aligned against the SILVA v128 database (BLAST threshold E value of *e*^−6^) for taxonomic annotation. After removal of the samples with small numbers of DNA reads, a total of 13,327,068 high-quality sequences and 2,422 OTUs were obtained for further analysis.

### Data statistics and analysis.

Alpha diversity indexes were computed using the diversity function in the vegan R package after random resampling. The differences in the PPE richness and proportions between the fresh samples and the preserved samples for each method were evaluated by *t* test using the t.test function in the stats R package. The storage time effect on the PPE abundance for different preservation methods was evaluated by one-way analysis of variance (ANOVA) using the aov function in the stats R package. The Kruskal-Wallis test was used to test the storage time (or preservation methods) effect on the Bray-Curtis dissimilarity of the fresh and F68 preservation samples using the kruskal.test function in the stats R package. The vegan package was used to analyze the community characteristics of the NMDS based on the Bray-Curtis distance. The Adonis test was used to test the PPE community differences between fresh and preserved samples using the adonis function in the vegan R package, based on Bray-Curtis dissimilarity. The PPE community differences between preservation methods were also evaluated by the Adonis test. To sort out the OTUs that were sensitive to aldehyde fixation, we divided the samples into two groups, i.e., the aldehyde group (FA, GA, FA plus F68, and GA plus F68 samples) and the no-aldehyde group (fresh and F68 samples). The R packages indicspecies and pheatmap were used to explore the indicator OTUs, whose relative abundances significantly changed between two groups (*P* < 0.05). All figures in this article were completed with the R package ggplot2 and Adobe Illustrator CS6.

### Data availability.

The raw sequencing reads have been deposited in the NCBI SRA under BioProject number PRJNA749152. R scripts and ancillary data are available at https://github.com/ChangqingLiu-CAS/Optimization-of-preservation-methods-provides-insights-into-photosynthetic-picoeukaryotes-in-lakes.
