# Supplementary material for: Optimization of Preservation Methods Provides Insights into Photosynthetic Picoeukaryotes in Lakes
Source: Microbiol Spectr. 2022 May 12;10(3):e02557-21. doi: 10.1128/spectrum.02557-21 (PMC9241741; doi:10.1128/spectrum.02557-21)
Supplement: SUPPLEMENTAL FILE 1 — Table S1, Fig. S1-S7. Download spectrum.02557-21-s001.pdf, PDF file, 1.5 MB [file spectrum.02557-21-s001.pdf]

**Supplemental material FOR publication**

Title: Optimization of preservation methods provides insights into photosynthetic picoeukaryotes in lakes

Authors: Changqing Liu, Jin Lei, Min Zhang, Fan Wu, Mingdong Ren, Jinsheng Yang, Qinglong Wu, and Xiaoli Shi

Supplementary Table 1 Taxonomic composition of PPEs retrieved from different lakes

Supplementary Fig. 1 The fluorescence image of tube wall at 400× magnification. (a) and (b) represent the unpreserved and preserved sample of  pure PPE culture, the dots with red fluorescence represent *M.homosphaera* cells.

Supplementary Fig. 2 The Cytogram of PC fluorescence versus Chl-a fluorescence from the fresh and preservation samples of artificial pond. P2 and P3 represent PPEs and picocyanobacterial, respectively.

Supplementary Fig. 3 NMDS analysis of the PPE community under the different preservation methods based on Bray-Curtis dissimilarity in each lake. Colors represent the storage time, and shapes represent the preservation methods.

Supplementary Fig. 4 Shared OTUs from water samples with different preservation methods and storage time.

Supplementary Fig. 5 Shared OTUs of PPE communities from water samples with different preservation methods and storage time.

Supplementary Fig. 6 The Cytogram of PC fluorescence versus Chl-a fluorescence from the fresh sample of artificial pond and the solutions of PBS buffer with different preservatives.

Supplementary Fig. 7 The agarose gel electrophoresis maps of sorted samples. E6, E7 and E8 represent the sorted samples from the negative controls. E9 represent the sorted samples without preservation. PC and M represent the positive DNA target control and DNA Marker of electrophoresis system, respectively.

**Supplementary Table**

Supplementary Table 1 Taxonomic composition of PPEs retrieved from different lakes

| Taxonomic affiliation | Lake Xuanwu | | Lake Chaohu | | Artificial pond | |
|---|---|---|---|---|---|---|
| | OTUs | Reads | OTUs | Reads | OTUs | Reads |
| **PPEs** | 123 | 213129 | 107 | 325618 | 143 | 90825 |
| Bacillariophyta | 11 | 95893 | 16 | 283244 | 25 | 22882 |
| Chlorophyta | 71 | 103875 | 56 | 29350 | 70 | 43262 |
| Chrysophyceae | 19 | 3656 | 16 | 7628 | 22 | 3178 |
| Dictyochophyceae | 8 | 3150 | 6 | 723 | 4 | 1462 |
| Dinophyceae | 2 | 2 | 2 | 23 | 2 | 1097 |
| Eustigmatophyceae | 1 | 1865 | 1 | 1190 | 1 | 24 |
| Haptophyceae | 2 | 4045 | 3 | 368 | 2 | 46 |
| Katablepharidophyta | 0 | 0 | 1 | 6 | 2 | 250 |
| Raphidophyceae | 0 | 0 | 0 | 0 | 0 | 0 |
| Synurophyceae | 6 | 350 | 5 | 3037 | 14 | 18610 |
| Xanthophyceae | 3 | 293 | 1 | 49 | 1 | 14 |
| **Non-PPEs** | 124 | 24770 | 142 | 12789 | 186 | 108181 |
| Annelida | 0 | 0 | 0 | 0 | 1 | 1 |
| Apicomplexa | 1 | 1 | 0 | 0 | 0 | 0 |
| Arthropoda | 8 | 1089 | 6 | 379 | 6 | 29 |
| Ascomycota | 29 | 11811 | 24 | 5675 | 25 | 1650 |
| Basidiomycota | 22 | 2846 | 17 | 1204 | 21 | 551 |
| Blastocladiomycota | 0 | 0 | 0 | 0 | 0 | 0 |
| Bryozoa | 0 | 0 | 0 | 0 | 0 | 0 |
| Chaetognatha | 0 | 0 | 0 | 0 | 0 | 0 |
| Chordata | 4 | 3276 | 2 | 698 | 4 | 245 |
| Chytridiomycota | 5 | 309 | 7 | 2078 | 17 | 15408 |
| Cryptomycota | 0 | 0 | 0 | 0 | 3 | 51 |
| Euglenida | 0 | 0 | 0 | 0 | 0 | 0 |
| Gastrotricha | 0 | 0 | 0 | 0 | 0 | 0 |
| Mollusca | 1 | 37 | 0 | 0 | 0 | 0 |
| Mucoromycota | 0 | 0 | 0 | 0 | 2 | 14 |
| Nematoda | 1 | 1 | 0 | 0 | 0 | 0 |
| Platyhelminthes | 0 | 0 | 0 | 0 | 0 | 0 |
| Porifera | 0 | 0 | 0 | 0 | 0 | 0 |
| Rotifera | 1 | 10 | 1 | 113 | 2 | 51 |
| Streptophyta | 16 | 4451 | 9 | 313 | 10 | 1484 |
| Zoopagomycota | 0 | 0 | 0 | 0 | 0 | 0 |
| Unclassified | 36 | 939 | 76 | 2329 | 95 | 88697 |
| **Sum** | 247 | 237899 | 249 | 338407 | 329 | 199006 |

**Supplementary Figure**

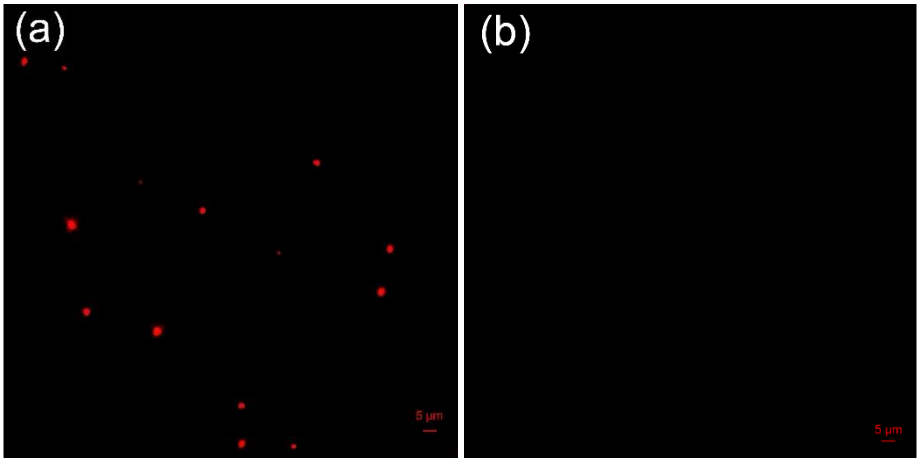


Supplementary Fig. 1 The fluorescence image of tube wall at 400× magnification. (a) and (b) represent the unpreserved and preserved sample of pure PPE culture, the dots with red fluorescence represent *M.homosphaera* cells.

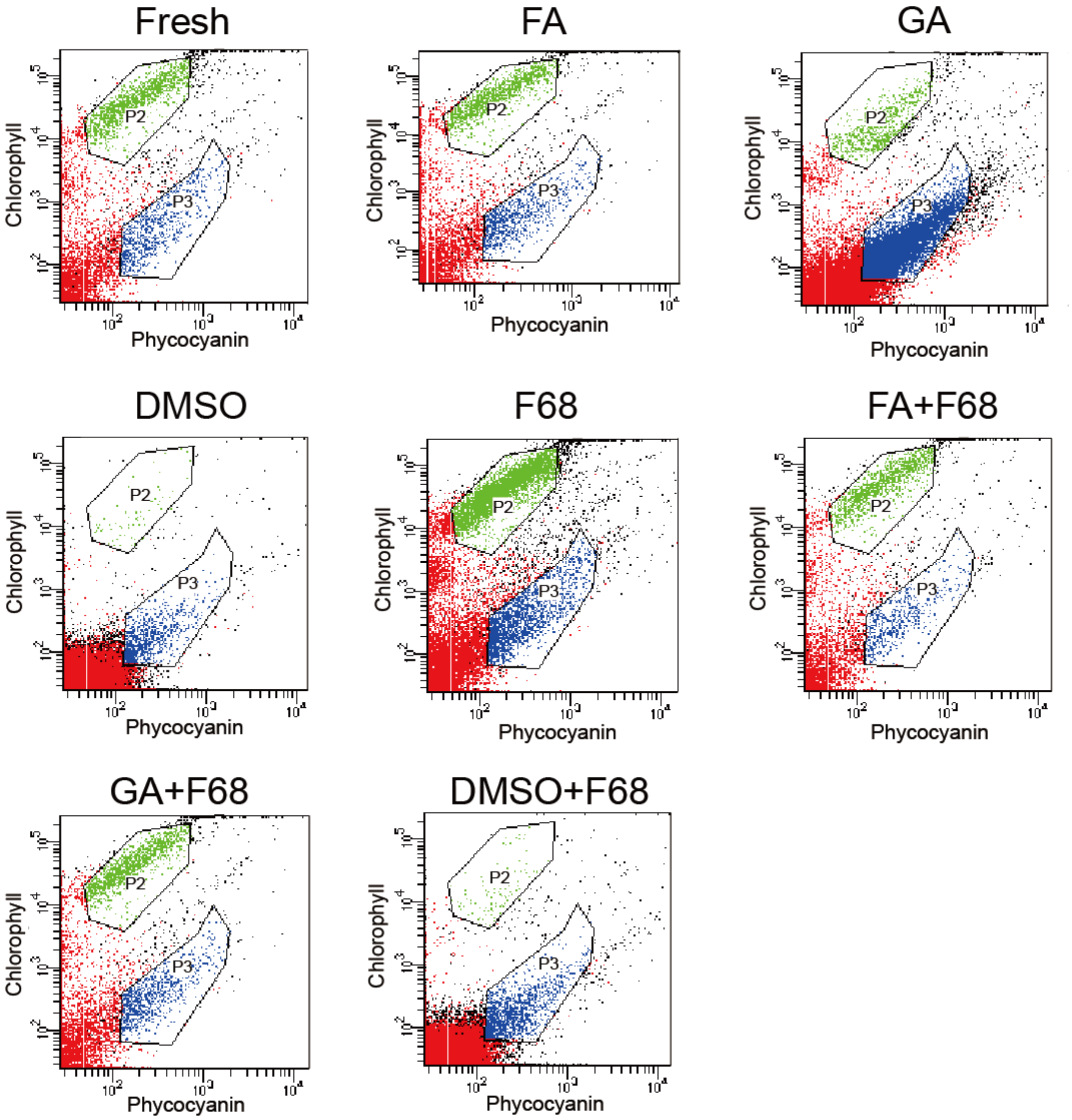


Supplementary Fig. 2 The Cytogram of PC fluorescence versus Chl-a fluorescence from the fresh and preservation samples of artificial pond. P2 and P3 represent PPEs and picocyanobacterial, respectively.

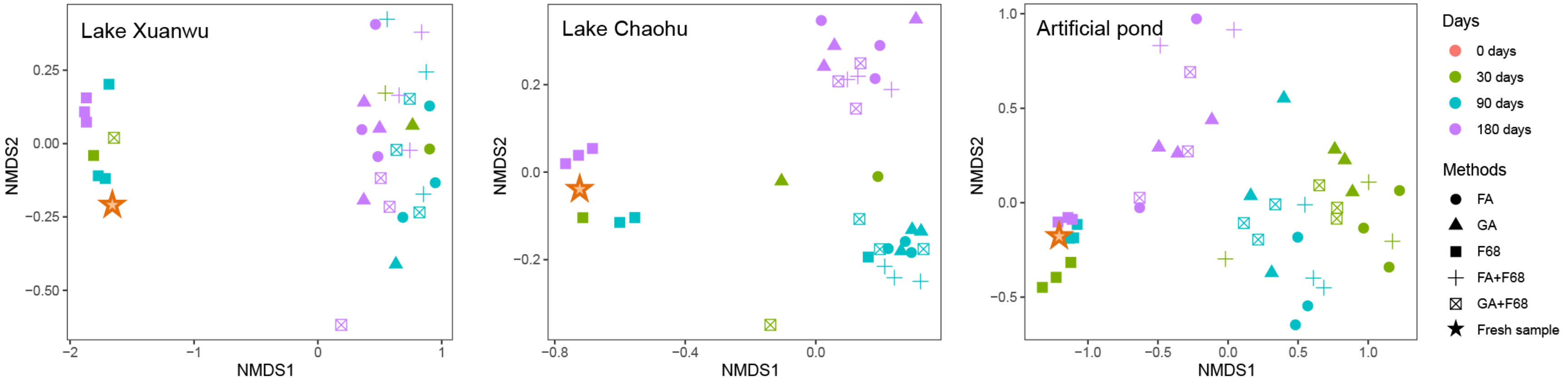


Supplementary Fig. 3 NMDS analysis of the PPE community under the different preservation methods based on Bray-Curtis dissimilarity in each lake. Colors represent the storage time, and shapes represent the preservation methods.

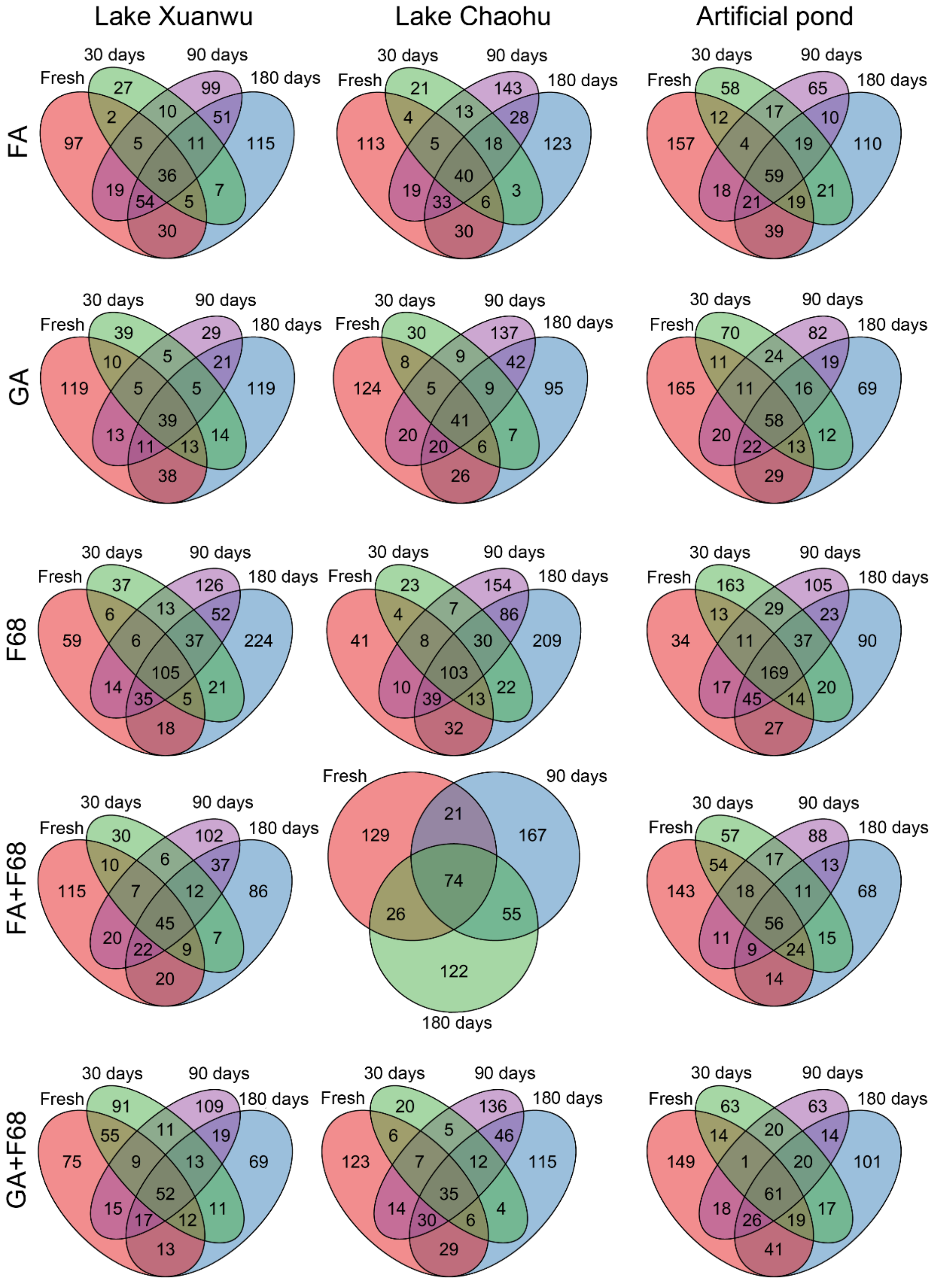


Supplementary Fig. 4 Shared OTUs from water samples with different preservation methods and storage time.

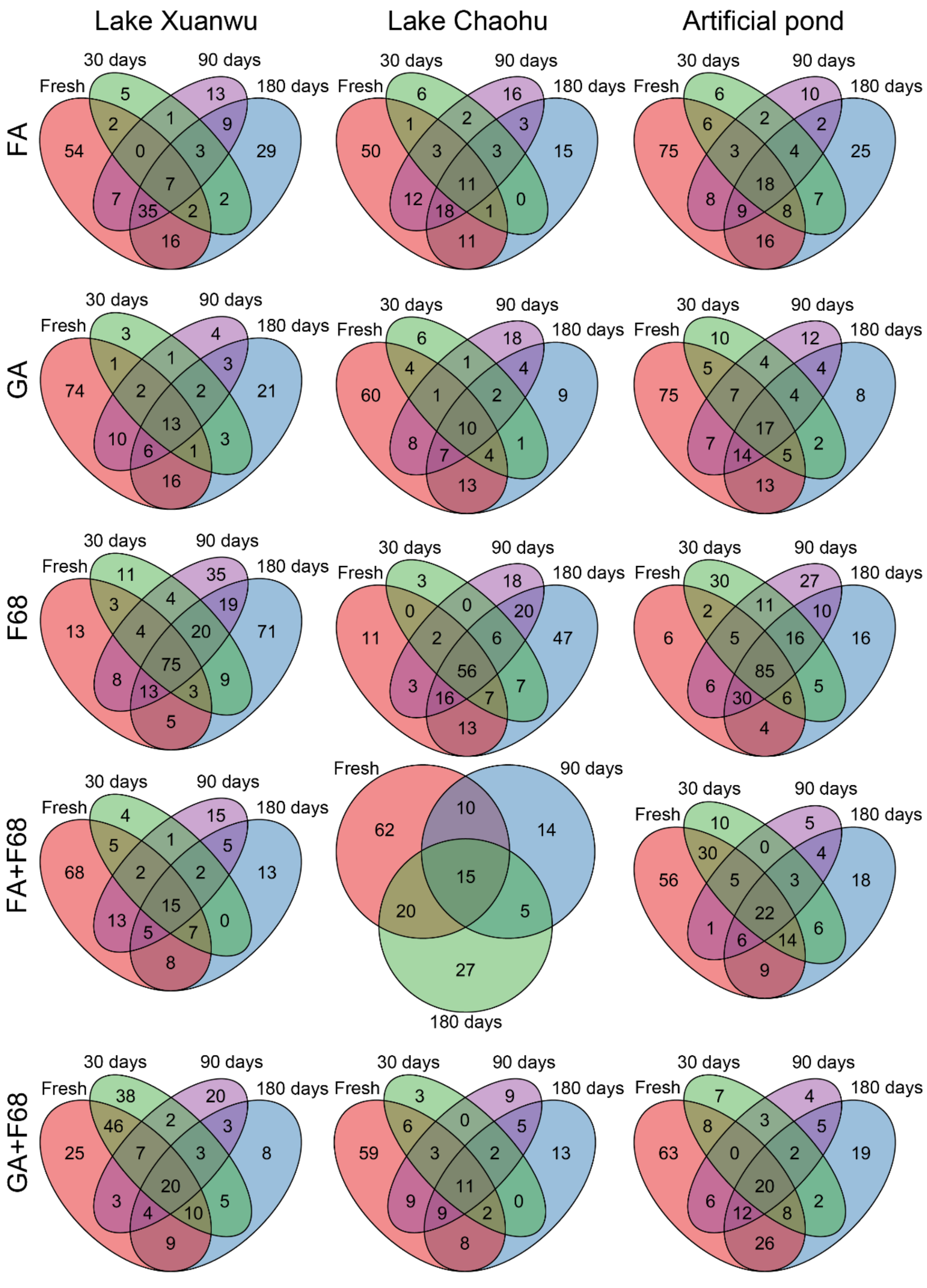


Supplementary Fig. 5 Shared OTUs of PPE communities from water samples with different preservation methods and storage time.

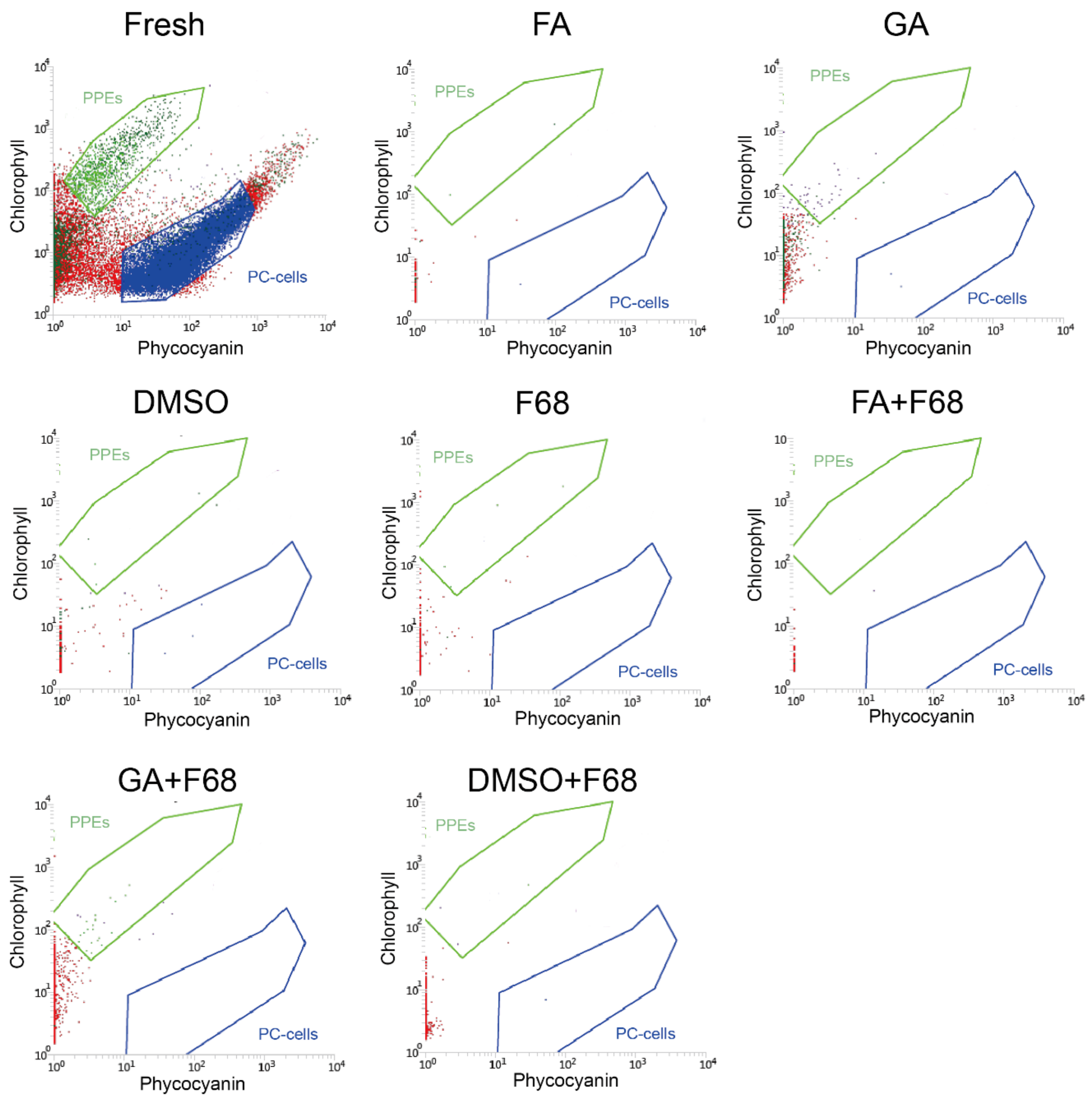


Supplementary Fig. 6 The Cytogram of PC fluorescence versus Chl-a fluorescence from the fresh sample of artificial pond and the solutions of PBS buffer with different preservatives.

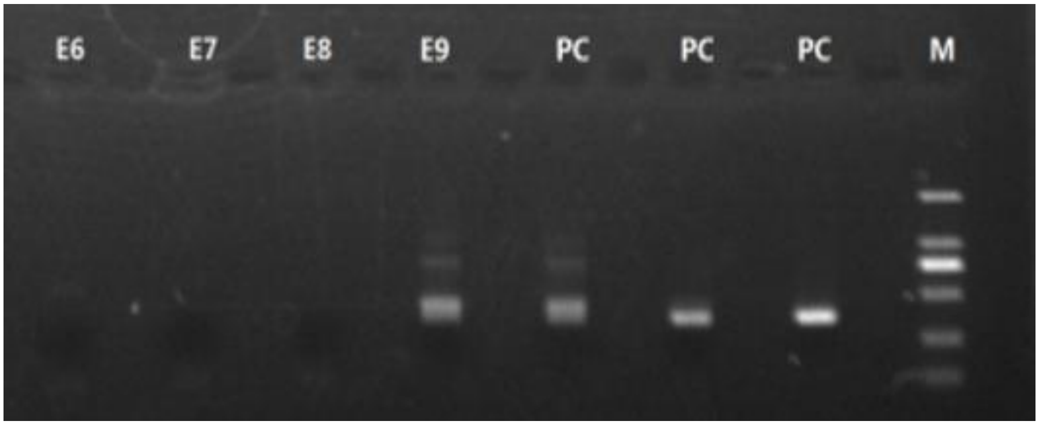


Supplementary Fig. 7 The agarose gel electrophoresis maps of sorted samples. E6, E7 and E8 represent the sorted samples from the negative controls. E9 represent the sorted samples without preservation. PC and M represent the positive DNA target control and DNA Marker of electrophoresis system, respectively.
